# Inhibitor of apoptosis proteins as therapeutic targets in bladder cancer

**DOI:** 10.3389/fonc.2023.1124600

**Published:** 2023-02-10

**Authors:** Philipp Wolf

**Affiliations:** ^1^ Department of Urology, Medical Center-University of Freiburg, Freiburg, Germany; ^2^ Faculty of Medicine, University of Freiburg, Freiburg, Germany

**Keywords:** bladder cancer, apoptosis, inhibitor of apoptosis proteins, cIAP1, cIAP2, XIAP, Survivin, Livin

## Abstract

Evasion from apoptosis is a hallmark of cancer. Inhibitor of apoptosis proteins (IAPs) contribute to this hallmark by suppressing the induction of cell death. IAPs were found to be overexpressed in cancerous tissues and to contribute to therapeutic resistance. The present review focuses on the IAP members cIAP1, cIAP2, XIAP, Survivin and Livin and their importance as potential therapeutic targets in bladder cancer.

## 1 Introduction

Bladder cancer (BC) is the 10th most common cancer worldwide with approximately 573,000 new cases and more than 212,000 deaths every year (1). Non-muscle invasive BC (NMIBC) is treated by transurethral resection of the tumor (TURBT) and intravesical adjuvant chemotherapy or Bacillus Calmette-Guerin (BCG) therapy. Patients with muscle-invasive BC (MIBC) are treated with neoadjuvant cisplatin-based chemotherapy or immunotherapy followed by radical cystectomy (RC). A bladder-sparing alternative option is the so called ‘trimodal therapy’, a regimen of maximal TRUBT followed by radiosensitizing chemotherapy and radiation. For patients with advanced or metastatic disease the median survival with standard chemotherapy is only about 13-15 months. Therefore, additional treatment options, such as immunotherapy with checkpoint inhibitors, targeted therapies with tyrosine kinase inhibitors or antibody drug conjugates are being tested [rev. in (2)]. Despite therapeutic progress in recent years BC is still characterized by a high recurrence rate and by a complex and expensive treatment with numerous local and systemic side effects. Evasion of apoptosis has increasingly been identified to contribute to tumor progression and resistance of BC and therefore targeted intervention in the apoptotic signalling pathways of BC cells might represent an improved treatment option for BC. The present review focuses on Inhibitor of Apoptosis Proteins (IAPs) and their importance as potential tumor targets in BC.

## 2 Inhibitor of apoptosis proteins

Apoptosis is a highly regulated and controlled genetic programme of cell death. It takes on an opposite role to mitosis and proliferation in the regulation of cell populations to ensure the correct embryonic development and to maintain the homeostasis of differentiated tissues in an adult organism (3, 4). It regulates the development of the nevous system and the immune system in that it eliminates surplus cells during formation of functional synaptic connections or productive antigen specificities, respectively. It is also responsible for the removal of pathogen-invaded cells and of inflammatory cells during wound healing [rev. in (4)]. It also plays a role in environmental toxicant-induced cell death, e.g after cell exposure to metals, dioxin or other cell-damaging chemicals (5).

**Figure 1:**
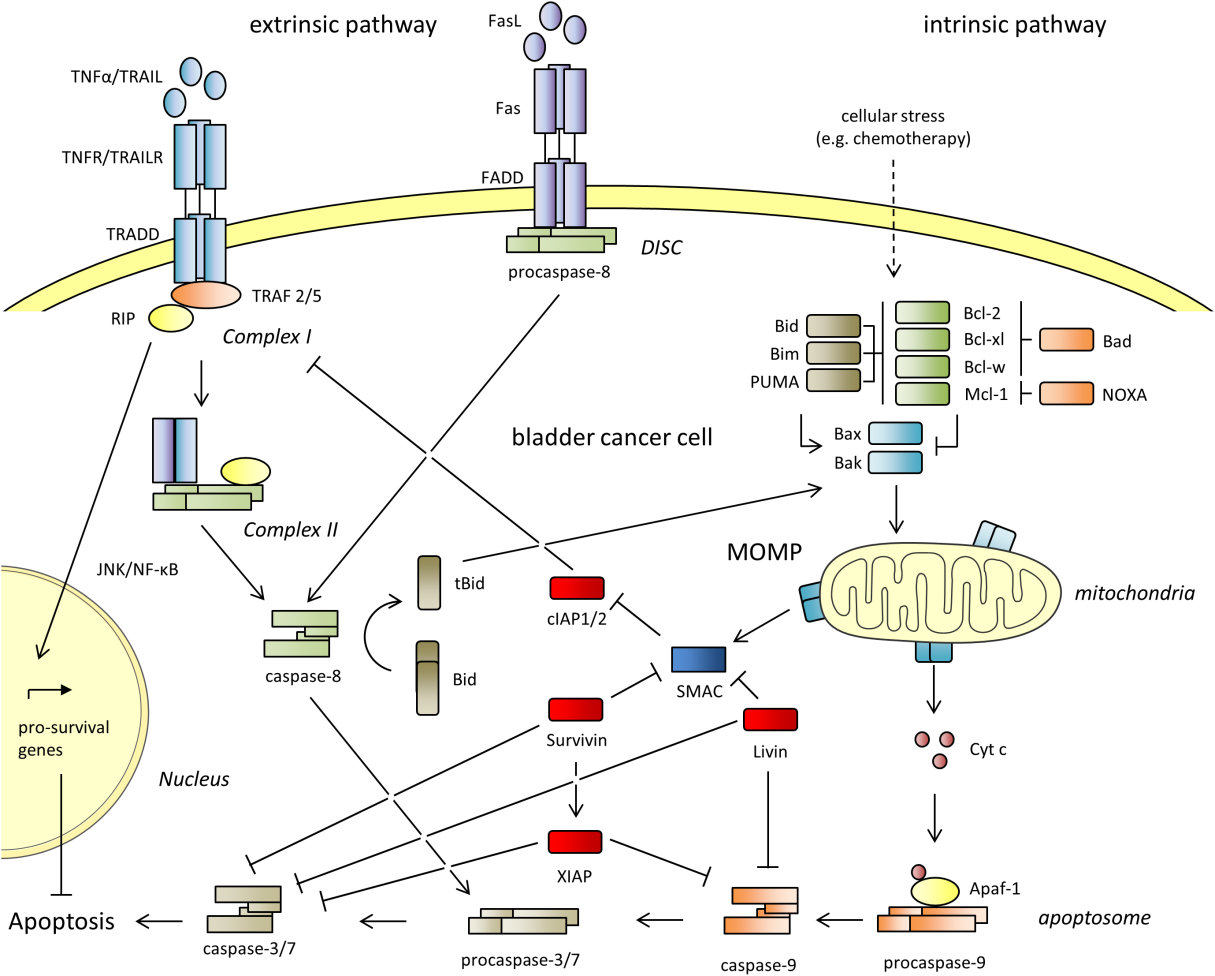
The extrinsic and the intrinsic apoptotic pathways. The Inhibitor of Apoptosis Proteins (IAPs) cIAP1 and 2, XIAP, Survivin and Livin are shown in red. They inhibit both pathways and lead to resistance against apoptosis. Apaf-1, apoptotic protease-activating factor 1; BAD, Bcl-2 antagonist of cell death; Bak, Bcl-2 antagonist/killer; Bax, Bcl-2 associated X protein; Bcl-2, B-cell lymphoma 2; Bcl-w, Bcl-2 like 2; Bcl-xl, B-cell lymphoma extra-large; BID, BH3 interacting domain death agonist; tBid, truncated Bid; BIM, Bcl-2 interacting mediator of cell death; Cyt c, cytochrome C; DISC, death-inducing signaling complex; FADD, Fas-associated protein with death domain; JNK, c-Jun N-terminal kinase; Mcl-1, myeloid cell leukemia sequence; MOMP, mitochondrial outer membrane permeabilization; NF-κB, nuclear factor kappa-light-chain-enhancer of activated B-cells; NOXA, phorbol-12-myristate-13-acetate-induced protein 1; PUMA, p53 upregulated modulator of apoptosis; RIP, receptor interacting protein; Smac, second mitochondria derived activator of caspases; TNF-α, tumor necrosis factor alpha; TRAIL, Tumor Necrosis Factor Related Apoptosis Inducing Ligand; TRADD, tumor necrosis factor receptor type 1-associated death domain protein; TRAF, TNF receptor associated factor.

Mammalian cells use the ‘extrinsic’ and the ‘intrinsic’ pathways to undergo apoptosis, which are shown in **Figure 1**. The ‘extrinsic’ pathway is engaged in response to extracellular signals and is initiated by binding of death receptor ligands (TNF-α, FasL, TRAIL) to their corresponding death receptors (TNFR, Fas, TRAILR). The binding of FasL to Fas initiates the recruitment of Fas-associated death domain (FADD) and procaspase-8 to form the death-inducing signaling complex (DISC), which in turn activates caspase-8 and the downstream effector caspases-3 and -7. The binding of TNF-α to TNFR or TRAIL to TRAILR initiates the recruitment of the adaptor protein TRADD, the Receptor-Interacting Protein (RIP) and and the TNF receptor associated factors (TRAF2/5) to form complex I. TRADD and RIP then dissociate from the receptors and associate with procaspase-8 and FADD to form complex II, which leads to the activation of the caspases -8, -3, and -7 for the execution of cell death. The ‘intrinsic’ pathway is triggered by environmental or intracellular stimuli, and leads to a dysregulation of pro- and anti-apoptotic B-cell lymphoma 2 (Bcl-2) proteins. When apoptosis is induced, the proapoptotic activators (Bid, Bim and PUMA) and sensitizers (Bad, NOXA) bind the anti-apoptotic proteins (Bcl-2, Bcl-xl, Mcl-1, Bcl-w) to free the effectors Bak and Bax. Additionally, the activators can directly activate both effectors. Bax and Bak then homo-oligomerize and form pores in the outer mitochondrial membrane. This culminates in mitochondrial outer membrane permeabilization (MOMP) and release of the proteins Cytochrome c (Cyt c) and the second mitochondrial activator of caspases (SMAC/Diablo) into the cytoplasm. Cyt c, the apoptotic protease-activating factor 1 (Apaf-1) and procaspase-9 then form the apoptosome, that facilitates the activation of caspase-9, -3 and -7. The extrinsic pathway can be reinforced by the intrinsic pathway by the so-called mitochondrial amplification loop. Mechanistically, Bid can be cleaved by caspase-8 to tBid. tBid can interact with Bax leading to its insertion into the mitochondrial membrane for the induction of MOMP. Moreover, the extrinsic and intrinsic apoptotic pathways are interconnected by sharing the effector caspases -3 and -7.

**Figure 2:**
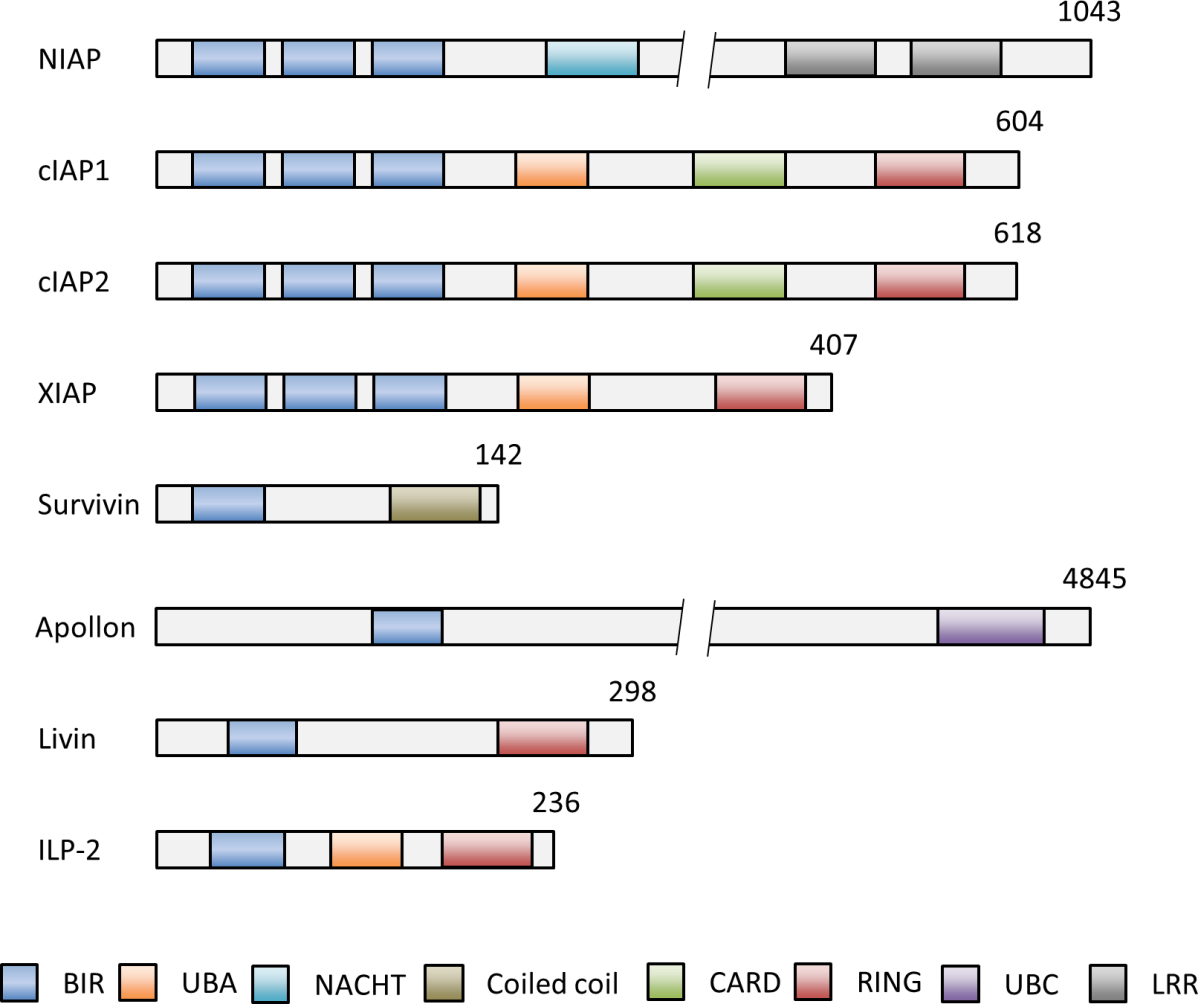
Schematic representation of the domain structures of the IAP members NIAP (BIRC1), cIAP1 (BIRC2), cIAP2 (BIRC3), XIAP (BIRC4), Survivin (BIRC5), Apollon (BIRC6), Livin (BIRC7) and ILP-2 (BIRC8). Numbers indicate numbers of amino acids of each IAP. The baculovirus IAP repeat (BIR) domain can bind and inhibit caspases. The, really interesting new gene (RING) domain has E3 ligase activity and for (auto-) ubiquitination of proteins for subsequent proteasomal degradation. The caspase activation and recruitment domain (CARD) domain mediates the autoinhibition of the E3 ubiquitin ligase activity by preventing RING dimerization. Other functional domains of IAPs comprise the ubiquitin associated domain (UBA) for binding to polyubiquitin chains for cell survival, activation of NF-kB signaling and proteasomal degradation, the ubiquitin conjugation domain (UBC) with E2 ubiquitin conjugating ligase activity and the neuronal apoptosis inhibitory protein domain (NACHT) with nucleoside-triphosphatase (NTPase) activity. BIR, aculorvirus IAP repeat; CARD, caspase recruitment domain; LRR, leucine-rich repeat; NACHT, neuronal apoptosis inhibitory protein; UBA, ubiquitin-associated.

The inhibitor of apoptosis proteins (IAPs) suppress apoptosis in both apoptotic pathways. IAPs were first identified in baculoviruses, DNA viruses that exclusively infect invertebrates, and found to block apoptosis in baculovirus infected insect cells (6). To date, eight different IAP family members were described in humans, called neuronal apoptosis inhibitory protein (NAIP, BIRC1), cIAP1 (BIRC2), cIAP2 (BIRC3), XIAP (BIRC4), Survivin (BIRC5), Apollon (BIRC6), Livin (BIRC7) and IAP-like protein 2 (ILP2, BIRC8) (**Figure 2**) (7).

The critical motifs required for the anti-apoptotic activity of all IAPs are the highly conserved baculovirus IAP repeat (BIR) domains (**Figure 2**) (8). The BIR domains are zinc-finger domains composed of a series of 4-5 α-helices and three β-strands and of three conserved cysteines and one conserved histidine, which coordinate a zinc ion (9). IAPs can bind with help of their BIR domains to caspases. This prevents the caspase interaction with cellular substrates and the proteolytic degradation of the cells as the final execution stage of apoptosis (10). Another significant domain is the ‘really interesting new gene’ (RING) domain in some IAPs, which acts as an E3 ubiquitin ligase. E3 ubiquitin ligases transfer the polypeptide ubiquitin to target proteins for subsequent proteasomal degradation (11). A few IAPs possess additional domains, such as the caspase activation and recruitment domain (CARD) (11, 12), the ubiquitin associated domain (UBA) (13), the ubiquitin conjugation domain (UBC) (14) and the neuronal apoptosis inhibitory protein domain (NACHT) (**Figure 2**) (15). Overall, IAPs can modulate the apoptotic pathways with help of their structural domains. They inhibit apoptosis by direct or indirect caspase inhibition and by facilitating the ubiquitination and proteasomal destruction of bound caspases. Moreover, rapid changes in IAP expression - controlled by (auto-)ubiquitination and proteasome-dependent destruction - can trigger apoptosis and reinforce the response to death-inducing stimuli (16–18). Presence and regulation of IAPs in a cell are thus decisive factors whether apoptosis can be induced or not.

## 3 IAPs as therapeutic targets in bladder cancer

Evasion of apoptosis is a hallmark of cancer and responsible for tumor development, progression and therapeutic resistance (19). It is based on a dysregulation of the apoptotic pathways, which includes the downregulation or inactivation of pro-apoptotic proteins as well as the upregulation or activation or anti-apoptotic proteins (20). IAPs were found to be frequently upregulated in cancer cells and can therefore serve as targets for cancer therapy (21).

In recent years, the expression and functional relationships of cIAP1, cIAP2, XIAP, Survivin and Livin in apoptosis of BC have been unraveled and are summarized in the following sections.

### 3.1 cIAP1/2 in BC

cIAP1 and cIAP2 are paralog proteins with a size of 618 aa and 604 aa, respectively (22). cIAP1 is abundantly present in almost all tissues. In contrast, cIAP2 was only found to be highly expressed in immune cells and weakly expressed in small intestine, kidney and lymphoid tissue (Human Protein Atlas proteinatlas.org) (23, 24). cIAP1/2 contain three BIR domains that facilitate direct binding to caspases and the RING domain with E3 ubiquitin ligase activity. For the prevention of cell death, cIAP1 is transported with help of the transport receptor CRM1 from the nucleus into the cytoplasm. There, it ubiqutinates RIP and other components of complex I. It thus prevents the formation of the complex required for the execution of apoptosis. Instead,the JNK and NF-κB signaling pathways are activated that induce the expression of a number of pro-survival genes, including some of the anti-apoptotic *Bcl-2* proteins, s*urvivin* and *cIAP2* (**Figure 1**) (25, 26).

cIAP1 expression was detected in 85% of BC samples and in 58% of normal bladder urothelium samples (27). Che and colleagues examined the subcellular distribution of both proteins and found a significantly enhanced expression of cytosolic cIAP1 (cIAP1-C), nuclear cIAP1 (cIAP1-N) and cytosolic cIAP2 (cIAP2-C) in BC compared to normal bladder urothelium. A significant correlation between cIAP1-N expression and tumor stage (MIBC vs. NMIBC; p=0.03) as well as tumor grade (low vs. high; p=0.01) was determined. Moreover, BC patients with a high cIAP1-N expression showed a shorter mean OS than patients with a low cIAP1-N expression (45.6 vs. 62.7 months, p<0.01) and a shorter mean recurrence-free survival (RFS, 30.1 months vs. 44.2 months, p<0.01) (28). Nuclear cIAP1 expression is therefore discussed as a prognostic factor for BC.

### 3.2 XIAP in BC

XIAP is a 497 aa protein and contains three BIR domains for effective binding of the active caspases-3, -7 and -9 (22). Moreover, the RING domain with E3 ubiquitin ligase activity leads to autoubiquitination and degradation of the caspases-3 and -7 (29). XIAP is ubiquitously expressed in human normal tissues (Human Protein Atlas proteinatlas.org) (23, 24) and overexpressed in many cancer cell lines and cancerous tissues (30). XIAP expression was detected in 66% of BC samples and in 36% of normal urothelium samples by immunohistochemistry (27). Li et al. observed XIAP expression in 61.4% (108 of 176) of patients with primary superficial BC treated with TURBT. No significant correlation between XIAP expression and tumor grading or staging was detected (p>0.05). Eighty-two (46.6%) patients experienced tumor recurrence at a mean time of 26.8 months. In 66 of them (80.5%) tumors were found to be XIAP-positive and in 16 of them (19.5%) to be XIAP-negative. Twelve patients with a XIAP-positive tumor, but no patient with a XIAP-negative tumor developed invasive disease at the time of relapse (p=0.0015) (31). Recently, Jin and colleagues found a molecular mechanism explaining the correlation between increased XIAP expression and metastasis in BC cells. In the highly metastatic BC cell line T24T they observed a downregulation of the transcription factor p-CREB, which resulted in a decreased expression of the micro RNA miR-200c. Decreased miR-200c led to an enhanced stabilization of XIAP mRNA, followed by XIAP overexpression and enhanced tumor cell invasion and metastasis (32).

XIAP seems also to play a role in chemoresistance of BC. *In vitro* assays with BC cell lines revealed a positive correlation of XIAP and mitomycin (MMC) resistance (33).

#### 3.2.1 Targeting cIAP1/2 and XIAP in BC

A classical method to inhibit cIAP1/2 and XIAP is the use of so-called SMAC mimetics, small peptides, which mimic the potent IAP antagonist SMAC (34). SMAC is released into the cytosol after MOMP and a 55 aa sequence is proteolytically cleaved to expose the N-terminal alanine–valine–phenylalanine–isoleucine (AVPI) motif, which can directly bind to a well-defined surface groove of the BIR3 domain in cIAP1, cIAP2 and XIAP. This prevents the IAPs to bind to caspases (35). SMAC mimetics basically contain the AVPI motif of SMAC to inhibit cIAP1, cIAP2 and XIAP or to induce their auto-ubiquitination for subsequent proteasomal degradation, which lowers the threshold for apoptosis in cancer cells (34). SMAC mimetics are therefore used in combination with drugs that stimulate the extrinsic (e.g. TRAIL, TNFα) or the intrinsic (e.g. chemotherapeutics) apoptotic pathways to enhance cancer cell death (36, 37).

In BC, SMAC expression was found to be downregulated compared to normal bladder urothelium. Whereas all normal bladders expressed SMAC, only 76% of BC samples expressed the protein. Remarkably, 98% of superficial BC samples (Ta and T1) were found to be SMAC positive, but only 41% of MIBC samples. Patients with SMAC-positive superficial BC had a longer RFS than those with lower SMAC expression after TURBT (p<0.05) and disease-specific survival of patients with invasive BC after radical cystectomy correlated positively with SMAC expression (p<0.05). Mizutani et al. demonstrated that SMAC expression is associated with chemosensitivity (38). This shows that the decreased expression of the IAP antagonist SMAC in BC may not only be used as marker for poor prognosis or chemoresistance. It also means that the IAPs cIAP1/2 and XIAP are less inhibited in advanced BC and that this condition can be overcome by administration of SMAC mimetics.

Wang and colleagues generated the SMAC mimetic SMACN7, which inhibited cell proliferation and induced apoptosis in T24 BC cells and sensitized them for MMC treatment (39). Another SMAC mimetic, called AZ58, led to a reduction of cIAP1, cIAP2 and XIAP expression, sensitized different BC cell lines to gemcitabine and cisplatin, and caused inhibition of tumor growth in a UMUC-6 mouse xenograft model (40). In a recent study, gemticabine- and cisplatin-resistant RT112 BC cells were verified to have enhanced cIAP1/2 and XIAP expression and to be cross-resistant to TRAIL-mediated apoptosis. Combination treatment with TRAIL and the SMAC mimetic LCL-161 resulted in an increased cytotoxicity compared to monotherapies. Whereas enhanced expression of cIAP1/2 and XIAP was noted after TRAIL treatment, presumably as a response to the pro-apoptotic stimulus, a reduced expression was determined after combination treatment with TRAIL and LCL-161 (41). In a recent study, the SMAC mimetic ASTX660, also known as Tolinapant, was found to induce necroptosis in apoptosis-insensitive BC cells by turning TNF-α into a cytotoxic signal (42). Like apoptosis, necroptosis is also a form of programmed cell death and can be induced by TNF-α. In brief, after binding of TNF-α to TNFR, a signaling complex, also called necrosome, is formed containing the proteins RIP1 and RIP3. RIP3 then phosphorylates and promotes the oligomerization of the kinase Mixed Lineage Kinase domain Like pseudokinase (MLKL). Oligomerized MLKL can then form pores in the plasma membrane leading to membrane damage and cell death [rev. in (43)].

Jinesh and colleagues found that the SMAC mimetic compound-C was able to sensitize BC cells to BCG-stimulated neutrophils, which constitute the main type of immune cells recruited in response to BCG treatment to release TNF-α, TRAIL and FasL. Compound-C caused decreased cIAP2 stability and reduced XIAP expression in the BC cells. Moreover, TNF-α was identified as the primary mediator of the cell death program triggered by BCG-stimulated neutrophils in combination with compound-C (44). Treatment of BC cells with SMAC mimetics might therefore be a promising therapeutic option to overcome BCG resistance.

### 3.3 Survivin in BC

Survivin is the smallest member of the IAP family. The 16.5 kDa protein contains a single BIR domain and a α-helical coiled-coil domain at the C-terminus. Survivin was found to inhibit the effector caspases -3 and -7 and to interact with XIAP for enhanced XIAP stability (45). Survivin is basically not expressed in normal tissues. Only a weak nuclear expression is seen in the squamous epithelium, in lymphoid tissues, testes and in the gastrointestinal tract (Human ProteinAtlas proteinatlas.org) (23, 24). In contrast, a high Survivin expression was detected in numerous hematological and solid tumors (46). Therefore Survivin is considered to be highly tumor-specific.

Survivin was not detected in the normal bladder urothelium, but in BC cells. In the study of Chen and colleagues, 74% of tissues from 105 patients after TURBT (NMIBC and MIBC) were found to be Survivin positive, whereas lack of expression was shown in 36 normal urothelium samples (27). In another study, Survivin was present in 64% of the BC samples and in 94% of lymph node metastases. In this cohort, Survivin expression was associated with disease recurrence (p=0.040), disease-specific mortality (p=0.037), and mortality (p=0.044) (47). Wang et al. detected a high expression of Survivin in 71% of NMIBC samples (48). Xi and colleagues found a comparable rate of 84.7%. In cases of recurrence, the high-expression rate was 98.3% (p<0.01) (49). Survivin was determined to be a marker of PFS (p=0.012) and to be a predictor for OS (p<0.001) in patients with T1 high grade BC (50). A recent study confirmed Survivin as a prognostic marker in BC for OS (P=0.008), PFS (P=0.025) and cisplatin resistance (51).

Due to its high tumor specificity, Survivin is a well known diagnostic urinary biomarker for BC. Noninvasive analysis of biomarkers in the urine is done with the aim to detect BC and recurrences while avoiding unnecessary biopsies. For example, Survivin RNA was significantly enriched in urine samples of BC patients at the time of diagnosis and led to an enhanced sensitivity of 68.8%, which was significantly higher than that of urine cytology (22%, p< 0.001). Remarkably, sensitivity was increased to 100% when survivin was combined with the markers Telomerase reverse transcriptase (hTERT) and Keratin (KRT7) (52). Although the usefulness of urinary biomarkers for detecting BC is highly controversial (53, 54), a new meta-analysis found that survivin serves as the best negative predictor to date (55).

#### 3.3.1 Targeting Survivin in BC

The data show that Survivin represents an excellent tumor specific target in BC. In recent years different therapeutic approaches were therefore developed to reduce the anti-apoptotic effects of Survivin, including siRNA, histone deacetylase (HDAC) inhibitors, antibiotics and Survivin inhibitors.

siRNA knockdown of Survivin was found to significantly reduce the viability of BC cells and to sensitize them to chemotherapy (56). Intratumoral treatment of mice bearing subcutaneous UM-UC-3 tumors with Survivin siRNA nanoparticles resulted in a 75% decrease in Survivin expression and 65% reduction of tumor volume (57). In another study Survivin siRNA encapsulated in liposomes reduced BC cell growth and significantly reduced the volume of orthotopically growing KU-7-luc derived tumors in nude mice after intravesical application (58).

In a recent study, combination of the HDAC inhibitor chidamide with MMC led to synergistic cytotoxicity in BC cells. Mechanistically, a downregulation of histone H3 expression and increase of its acetylation was found followed by suppression of the Axl pathways and its downstream molecules clapsin and Survivin (59).

Lin and colleagues recently identified nitroxoline, an antibiotic for the treatment of urinary tract infections, as a new signal transducer and activator of transcription 3 (STAT3) inhibitor. The antitumor effects of nitroxoline in chemoresistant BC cells were based on the downregulation of STAT3 signaling followed by downregulation of the anti-apoptotic proteins Bcl-xl, Mcl-1 and Survivin, among others (60).

The survivin inhibitor sepantronium bromide (YM155) was tested against different human cancer cell lines and xenograft models. A concentration dependent inhibitory activity of YM155 was detected in a panel of different BC cells and in a UM-UC-3 mouse xenograft model (61). YM155 was also used to overcome gemcitabine resistance of BC cells (62). The recently developed Survivin inhibitor LQZ-7I targets the dimerization interface of Survivin. Inhibition of dimerization causes the misfolding of Survivin followed by proteasomal degradation. LQZ-7I was successfully used to downregulate Survivin in prostate cancer cells and to induce apoptosis *in vitro*. Moreover, the inhibitor led to a reduction of prostate tumor growth in a nude mouse xenograft model after oral administration (63). Future studies will demonstrate whether this inhibitor is also effective in BC.

### 3.4 Livin

Livin is a 32.8 kDa protein. Direct interactions between the BIR domain of Livin and SMAC, caspase -9, -3 and -7 were detected (64, 65), because it has structural similarity with the BIR3 domain of XIAP (66). Interactions of Livin with the pro-apoptotic proteins prevented apoptosis induced by death receptors and chemotherapeutic agents (67). Livin seems to degrade SMAC with help of its E3 ubiquitin ligase activity, for which both the BIR and RING domains are required. Moreover, the RING domain promotes auto-ubiquitination leading to a short half-life of Livin of less than four hours (68).

Like Survivin, Livin is not present in most normal tissues. It is only found in some cells of the squamous epithelium, in lymphoid tissues, testes and in the gastrointestinal tract (Human ProteinAtlas proteinatlas.org) (23, 24). A high Livin expression, however, was detected in several cancers, including leukemia, lymphoma, melanoma, colon cancer, breast cancer, cervix cancer, stomach cancer, prostate cancer, and NSCLC (69).

There are two Livin isoforms, named Livin α and β, which differ in a 54 bp deletion in exon 6. Until today, only Livin α has been reported to be expressed in BC (70, 71). Livin is suggested to be involved in the progression of superficial BC (71)and showed a high expression level in 65% of NMIBC tissues (48). In another study, Livin was detected in 39% of tissues from 105 patients after TURBT (NMIBC and MIBC), whereas lack of expression was shown in the normal urothelium (27). Xi et al. detected Livin in 75.0% of 72 NMIBC samples. In cases of recurrence, a high-expression rate of 91.7% was found (P < 0.01) (49).

#### 3.4.1 Targeting Livin in BC

The role of Livin as a therapeutic target in BC is largely unknown. Yang and colleagues used siRNA for Livin knockdown and found inhibition of proliferation and colony formation in T24 cells. Furthermore, the Livin knockdown resulted in an enhanced apoptosis rate in response to stimuli of the extrinsic (TNF-α) and intrinsic (MMC) apoptotic pathways (70). Recently, a Livin inhibitor, called 142I5 (compound 7), was developed, which binds to the BIR domain of Livin, and also to the BIR3 domains of cIAP1, or cIAP2 and XIAP with weaker affinity. The inhibitor significantly increased apoptosis in the melanoma cell line SK-MEL-28 in combination with TNF-α in a dose- and time dependent manner (67). We are ongoing to test 142I5 in BC cells to see if it can also induce increased apoptosis in BC cells.

## 4 Discussion

In the last years, IAPs were found to be overexpressed in BC and to be associated with therapeutic resistance. Individual IAP members have been identified as suitable diagnostic and prognostic markers for different subgroups of BC. Moreover, an inhibition of IAPs in BC led to downregulation of the apoptotic threshold for apoptotic stimuli like TRAIL/TNF-α or chemotherapy. Drugs that interfere with the effects of IAPs could therefore be advantageous in future to support intravesical treatment of NMIBC patients with BCG or MMC and to reduce the rate of recurrence. They could also enhance the sensitivity of BC cells for adjuvant or neoadjuvant chemotherapy of patients with MIBC.

A key point in future is to establish appropriate investigational models in order to assess the efficacy of IAP targeting in BC patients.Targeting of IAPs has been previously studied in established BC cell lines and in derived *in vivo* tumor xenograft models. A problem is, that there is a predominance of more aggressive tumor subtypes among these cell lines. This means that testing drugs for targeting IAPs in these cell lines might not recapitulate drug responses in BC patients, especially in those with lower tumor stages (72). Therefore, research is ongoing to generate new cell lines representing a broader panel of different BC stages and to establish patient-derived organoids and xenografts that recapitulate the histopathological and molecular diversity of human BC. Moreover, examination of individual IAP expression and testing of suitable inhibitors on patient-derived cells acquired from biopsies before and after recurrence could also help to generate individual therapies (73, 74).

Research is also still needed on the exact mode of action of IAP inhibition. Especially, SMAC mimetics for the inhibition of cIAP1/2 and XIAP have extensively been tested in preclinical studies and have entered clinical trials for the treatment of hematological and solid cancers - unfortunately with limited results so far. One major obstacle for a high efficacy of SMAC mimetics in the clinic is the ubiquitous expression of the targets cIAP1, cIAP2 and XIAP in normal tissues, which can cause side effects and can be dose-limiting. A second one can be found on the molecular level. Darding and colleagues discovered in HEK293T cell models that degradation of cIAP2 by SMAC mimetics depends on the presence of TRAF2 and cIAP1. It was suggested that TRAF2 builds a platform for the heterodimerisation of the RING domains of cIAP2 and cIAP2. The SMAC mimetics then stimulate the autoubiquitination and degradation of the cIAPs. In the absence of cIAP1 cells with *de novo* synthesized cIAP2 was resistant to SMAC mimetic treatment (75).

Since SMAC mimetics alone were found to have only a small therapeutic efficacy, a more detailed analysis of the expression patterns of IAPs at different BC stages may, in the future, lead to the simultaneous inhibition of multiple IAPs to achieve a better clinical outcome. Since IAP inhibition serves to lower the threshold for triggering the extrinsic or intrinsic apoptosis pathways, combinations of IAP inhibition with chemotherapy or radiation represent new treatment options and help to treat chemo- or radiation-resistant cancer cells [rev. in (36]. Moreover, induction of immunogenic apoptosis or necroptosis after treatment of cancer cells with IAP inhibitors in combination with TNF-α or radiation opens up new avenues in cancer therapy (42, 76). Immunogenic apoptosis and necroptosis represent different types of ICD (77). ICD is characterized by the release of damage-associated molecular patterns (DAMPs) and can induce an effective antitumor immune response through activation of dendritic cells (DCs) and consequent activation of a specific T cell response (77). Future preclinical studies are needed to characterize ICD in BC cells after IAP targeting, especially in apoptosis-resistant stages. Moreover, clinical trials using the SMAC mimetic BI 891065 for the targeting cIAP1, cIAP2, and XIAP in combination with the anti-PD1 antibody BI 754091 against solid tumors include the treatment of BC patients and will show whether the SMAC mimetics are helpful in the future immunotherapy of BC (NCT03166631, NCT04138823).

In contrast to cIAP1, cIAP2 and XIAP, Survivin and Livin are largely tumor-specific. The recent development of new specific inhibitors against these IAPs increases their importance as promising targets in the future treatment of BC. So far, nothing is known about the IAPs ILP-2, NAIP, and Apollon and their role in BC. Therefore, their expression and significance as therapeutic targets in BC should also be the focus of future research.

## Author contributions

The author confirms being the sole contributor of this work and has approved it for publication.
